# Effects of FSH on Sperm DNA Fragmentation: Review of Clinical Studies and Possible Mechanisms of Action

**DOI:** 10.3389/fendo.2018.00734

**Published:** 2018-12-11

**Authors:** Monica Muratori, Elisabetta Baldi

**Affiliations:** ^1^Department of Experimental and Clinical Biomedical Sciences “Mario Serio”, University of Florence, Florence, Italy; ^2^Department Experimental and Clinical Medicine, University of Florence, Florence, Italy

**Keywords:** testis apoptosis, DNA fragmentation, human spermatozoa, oxidative stress, follicle-stimulating hormone

## Abstract

Sperm DNA fragmentation (sDF) is an important reproductive problem, associated to an increased time-to-pregnancy and a reduced success rate in natural and *in vitro* fertilization. sDF may virtually originate at any time of sperm's life: in the testis, in the epididymis, during transit in the ejaculatory ducts and even following ejaculation. Studies demonstrate that an apoptotic pathway, mainly occurring in the testis, and oxidative stress, likely acting in the male genital tract, are responsible for provoking the DNA strand breaks present in ejaculated spermatozoa. Although several pharmacological anti-oxidants tools have been used to reduce sDF, the efficacy of this type of therapies is questioned. Clearly, anti-apoptotic agents cannot be used because of the ubiquitous role of the apoptotic process in the body. A notable exception is represented by Follicle-stimulating hormone (FSH), which regulates testis development and function and has been demonstrated to exert anti-apoptotic actions on germ cells. Here, we review the existing clinical studies evaluating the effect of FSH administration on sDF and discuss the possible mechanisms through which the hormone may reduce sDF levels in infertile subjects. Although there is evidence for a beneficial effect of the hormone on sDF, further studies with clear and univocal patient inclusion criteria, including sDF cut-off levels and considering the use of a pharmacogenetic approach for patients selection are warranted to draw firm conclusions.

## Introduction

FSH (follicle-stimulating hormone or follitropin) is the main hormone regulating the development and the functions of male and female gonads. It is a glycoprotein heterodimer consisting of two chains, α (92 amino-acids) and β (111 amino-acids) which are coupled by a non-covalent bond. The hormone acts by binding its receptor (FSHR) which belongs to the superfamily of the seven transmembrane domain G-protein-coupled receptors and is expressed in the gonads. After binding to FSHR, FSH activates the cAMP-protein kinase A cascade, which regulates gene expression through phosphorylation of CREB transcription factors [for a comprehensive review on FSR receptor signaling see (1)]. The action of FSH is influenced by the presence of both polymorphisms of FSHR, affecting the sensitivity of the receptors to the hormone (2), and the β chain of the hormone, which is associated with significantly lower serum FSH levels (3). FSHR and FSHβ polymorphisms influence the response to treatment with FSH in both women (4, 5) and men (6). In particular, in the adult testis, FSH regulates spermatogenesis by acting on Sertoli cells and there is evidence that FSHR polymorphisms are associated with male infertility (7).

FSH is essential for induction of qualitative and quantitative maintenance of spermatogenesis (8), as also demonstrated by studies on FSHR KO animals, which present severe disturbances of testicular function, including small testis and aberrant gametogenesis (9–11). Besides hypogonadotropic hypogonadic men (12), highly purified or recombinant FSH has been proposed for the treatment of infertile normogonadotropic men with idiopathic oligozoospermia or oligoasthenoteratozoospermia (OAT). In human, several trials using FSH to treat men with alterations of spermatogenesis, in particular OAT men, have been published. Although many of these studies report improvement of sperm parameters, such as concentration and motility, the efficacy of FSH treatment for OAT subjects remains controversial (6, 13). Even more controversy exists regarding the effect of FSH treatment on sperm morphology (14–17). Controversy may depend on heterogeneity of the study characteristics, in particular patient inclusion criteria (including FSH basal levels, FSHβ and FSHR genotypes), the dose and the molecule of administered FSH, the length of the treatment and the presence of non-responding men (18). Despite such controversy, a Cochrane meta-analysis (19) including only randomized control trials in which gonadotrophins were compared with placebo or no treatment, suggests a beneficial effect of FSH treatment on live birth and pregnancy after natural conception in men with idiopathic male factor subfertility, but no significant effects after assisted reproduction techniques (ARTs). A more recent meta-analysis (20) evaluating 15 controlled clinical studies [with broader inclusion criteria respect to (19)] with overall 614 men treated with FSH vs. 661 treated with placebo or untreated, confirms the improvement of spontaneous pregnancy and reveals a significant effect also after ARTs, which is independent on the ART methodology. Interestingly, 11 studies evaluated also sperm parameters after FSH treatment and the meta-analysis of these studies indicated that the treatment induced a significant increase of sperm concentration (although with a high degree of heterogeneity of the studies) and a trend to a better progressive sperm motility. However, a meta-regression analysis of the same studies showed no significant correlation between pregnancy rate and sperm parameters (concentration, progressive motility) (20) in line with previous studies demonstrating the poor predictive value of semen parameters for attainment of pregnancy (21, 22). Thus, the improvement of pregnancy rate following treatment of subfertile men with FSH is likely due to effects on other sperm qualities (such as sperm DNA fragmentation (sDF), see below) or on testicular functions leading to an improvement of sperm functions necessary for the process of fertilization which are not evaluated by routine semen analysis (such as hyperactivation motility, ability to undergo acrosome reaction or increased chromatin compaction). In this respect, a recent study (23), demonstrated that treatment with FSH improves the percentage of spermatozoa able to bind hyaluronic acid in FSH responding men (i.e., men increasing total sperm count and total motile sperm count after FSH treatment). As ability to bind hyaluronic acid is indicative of higher sperm maturation (24), the study by Casamonti et al. (23) suggests that FSH may improve such testicular function. Alterations in sperm maturation process are also involved in the generation of sDF (see below).

This review focuses on the effect of FSH administration to idiopathic infertile men on sperm DNA fragmentation levels, discussing the possible mechanisms involved in the action of the hormone.

### Sperm DNA Fragmentation (sDF)

The main function of spermatozoa is to deliver DNA to the oocyte at fertilization. Integrity of sperm and oocyte DNA is fundamental for development and quality of embryos. Sperm DNA integrity is often compromised in infertile men and sDF represents the most common DNA abnormality in these men (25). sDF consists in the presence of single and double DNA strand breaks in the sperm nucleus. Such breaks may occur at different levels of the sperm's life, virtually from early steps of spermatogenesis to the site of fertilization. Indeed, there is evidence that sperm DNA breaks may originate in the testis, in the epididymis, during transit in the ejaculatory ducts, following ejaculation and even during *in vitro* manipulation for ARTs. Many types of insults have been demonstrated to provoke DNA breaks, which act through two main pathways: an apoptotic process, leading to activation of endonucleases and a direct attack to DNA by free radicals which produces both base oxidation and strand breaks (26). The apoptotic process occurs mainly during spermatogenesis, either because of insults impairing the testicular function or because of a derailment of the chromatin condensation process during spermiogenesis (27, 28). Spermatozoa with apoptotic signs (including DNA breaks) are found in the ejaculate because the apoptotic process fails to complete [abortive apoptosis, (29)]. Although free radicals, at low levels, play an important role for sperm functions [such as motility and capacitation (30)], when ROS production overtakes the anti-oxidant defenses of spermatozoa several damages can be produced (31). Excessive ROS production may act virtually at any level during sperm's life (32), although evidence suggests that their action occurs mostly after spermiation (see below) and even during *in vitro* manipulations for ARTs (33, 34). The occurrence of defects in the process of chromatin compaction renders the spermatozoa particularly vulnerable to ROS attack (35). Muratori et al. (28) has recently reported that a clear overlapping between oxidative damage and DNA breaks was detected only in viable spermatozoa, whereas in the bulk of ejaculated spermatozoa (including viable and non-viable cells and where most DNA fragmented spermatozoa are non-viable) the presence of DNA breaks overlapped highly apoptotic traits. Considering that viable, DNA fragmented spermatozoa are cells where DNA damage developed more recently respect to the ejaculation (28), these results suggest that oxidative stress acts later in sperm's life, most likely during transit in the male genital tract, whereas apoptotic damage occurs earlier, mainly at testicular level. A recent clinical study (36) seems to confirm such hypothesis revealing that sDF in unviable spermatozoa is associated mainly with the presence of ultrasound signs of testicular abnormalities, whereas the DNA fragmented sperm population containing viable spermatozoa was mostly associated with clinical and ultrasound alterations of the prostate and of seminal vesicles, likely due to inflammatory statuses. There is also evidence that DNA damage may occur after ejaculation during *in vitro* incubations (37–39) or because of *in vitro* manipulation during sperm selection for ARTs (33, 34, 40). In the latter case, DNA fragmented spermatozoa are highly motile and the damage appears to be induced by the contamination with heavy metals of density gradient preparations (33). Viable sperm with oxidative damage and/or strand breaks in their DNA are, most likely, a very dangerous sperm fraction of the ejaculate: they can actively participate in the fertilization process and give rise to embryos unable to successfully develop if the oocyte does not or only partially repairs the damage.

Many studies (41–43) reported that high levels of sDF are associated with a decrease of natural male fertility and recent meta-analyses confirmed the negative relationship between the amount of sDF and the outcomes of natural or assisted reproduction (44–46). It should be noted that important differences exist among the studies on ART outcomes, especially regarding couple inclusion criteria and methods used to evaluate sDF. Indeed, sDF may be evaluated by several methods [reviewed in (47)], among which TUNEL (Terminal deoxynucleotidyl transferase dUTP nick end labeling), COMET (also known as single-cell gel electrophoresis), SCSA (Sperm Chromatin Structure Assay) and Halosperm assays are the most popular. The problem with these methods is that they likely detect different types of DNA damages (47). In addition, these methods (with the exception of SCSA) are not standardized, thus making difficult to compare results among the studies. Recent meta-analyses grouped the studies according to the methods used to evaluate sDF and reported consistently that TUNEL and COMET methods are those that better reveal the negative association between sDF and pregnancy rate after ARTs (45, 46). TUNEL also resulted the method that better reveals the impact of sperm DNA damage on miscarriage in couples who conceived naturally or after IVF and ICSI (44).

Overall, the bulk of the studies described above suggests that sDF represents a target to treat men with idiopathic infertility. In consideration that apoptosis and oxidative stress are the main mechanisms producing DNA strand breaks (see above) possible therapies to prevent or decrease sDF are antioxidants and anti-apoptotic agents. The former have been used in several clinical studies, but, so far, reported beneficial effects are minimal. Indeed, a recent Cochrane meta-analysis (48) could not draw definitive conclusions regarding the benefit of treatment with anti-oxidant on live birth rates for infertile couples as only four low quality small randomized controlled trials were published at that time. The same meta-analysis reported also data about the effect of antioxidants on sDF levels. Even in this case, no clear conclusions could be drawn because the two trials included in the meta-analysis utilized different antioxidants in a low number of patients (48). Use of anti-apoptotic agents, on the other hand, is not feasible because of the ubiquitous role of programmed cell death in the body. A notable exception is represented by FSH which has specific anti-apoptotic (or pro-survival) effects at testicular level (49–52).

### Effect of Treatment With FSH on sDF Levels

A recent meta-analysis evaluated the effect of FSH on SDF (53) including six studies with overall 383 men with idiopathic infertility treated with FSH. The meta-analysis revealed a slight but significant decrease of sDF after FSH treatment for 3 months but not of other semen parameters such as sperm concentration, motility and morphology. Of note, the studies included in the meta-analysis are extremely heterogeneous, both for inclusion criteria and FSH treatment scheme. Indeed, in three of them patients with severe oligozoospermia (54) or oligoasthenoteratozoospermia (15, 55) were included, in another (56), patients with at least one parameter below the WHO criteria, whereas in the paper by Garolla et al. (57) male partners of infertile couples with any kind of infertility cause (with exclusion of seminal tract infections and antisperm antibodies) were included if sperm count was above 20 millions. The only study where sDF basal levels (at the cut-off level >15%) were comprised in the inclusion criteria was that of Simoni et al. (6). Interestingly, Ruvolo et al. (55), demonstrated that patients with sDF levels >15% were those showing a significant reduction in DNA sperm damage. More recently, Colacurci et al. (58) published results of a multicentric longitudinal trial including 103 infertile men treated with FSH for 3 months: the study demonstrated a slight but significant effect of the hormone on average sDF levels. Interestingly, this study evidenced that the treatment was more effective in the 48 patients showing sDF levels above 17% (median value of the caseload) and demonstrated that lifestyle habits like smoking may decrease the effectiveness of the therapy. The clinical studies included in the meta-analysis of Santi et al. (53) were heterogeneous also regarding the treatment schemes (type and dosage of FSH used) and the methods used to evaluate sDF, even if most studies employed TUNEL assay (6, 15, 54, 55, 57). It should be considered that TUNEL is not a standardized method and it has been reported that even small variations in the different steps of the assay may affect greatly the measures (59). In addition, an important difference regards the detection method: TUNEL positive spermatozoa may be evaluated by flow cytometry in thousands of spermatozoa [as used in the papers by (57) and (6)] or by fluorescence microscopy in few hundreds of spermatozoa [used in (15, 54, 55)]. Discrepancies between the two detection methods are due not only to the different number of analyzed cells but also to the different sensitivity of the procedures. For these reasons, comparison of studies employing flow cytometry or fluorescence microscopy revealed that the former yields greater measures of sDF (60). This methodological issue can explain why the meta-analysis of Santi et al. (53) failed to find a difference in the average sDF levels after treatment when comparing FSH treated and untreated men.

There is also evidence, in the literature, that specific genotypes of FSHR (the polymorphism p.N680s) predicts responsiveness to FSH administration (6) and that the polymorphism of FSH beta-subunit promoter FSHB-211 TT is associated with lower FSH levels and lower sperm counts (61). Overall these studies suggest that the use of pharmacogenetic approaches to select patients, may increase the percentage of responders to the therapy.

Clearly, larger studies are needed to confirm the ameliorative effect of FSH on sDF: such studies should be properly designed, possibly using selection criteria which include a cut-off of sDF basal levels and the above mentioned pharmacogenetic approaches. However, it must be mentioned that, due to lack of international standardized procedures to evaluate sDF, identifying a cut-off value depends strictly on the assay used to measure the parameter. At present, the only possibility is the identification of cut-off values by comparing fertile and infertile subjects in each laboratory using the chosen method to evaluate sDF among those currently available (see above).

Which is(are) the mechanism(s) through which FSH ameliorates sDF levels in the ejaculate? If we consider that most DNA fragmented spermatozoa show signs of apoptosis and chromatin immaturity (28) likely due to a derangement of the spermatogenetic process or of the chromatin maturation process, the most probable mechanisms of action of FSH consist in anti-apoptotic and maturation promoting effects at tubular level. There is evidence of anti-apoptotic effects of FSH both in the ovary and in the testis. In the ovary, the hormone is a major survival factor for follicles (62) and antagonizes the apoptosis induced by oxidative stress reducing ROS production through stimulation of the antioxidant glutathione (GSH) (63). In the testis, suppression or immunoneutralization of FSH increases apoptotic DNA fragmentation (49–51, 64). FSH suppression induces spermatogonial apoptosis predominantly via the intrinsic pathway, as an increase of caspase activity (52) and a decrease of BCL2 (51) have been demonstrated in spermatogonial cells. Consistently, *in vitro* studies demonstrated up-regulation of the BCL2 family member Bcl2l2 mRNA in spermatogonia of adult mice after FSH treatment (65). However, the molecular details by which FSH deprivation leads to activation of the apoptotic intrinsic pathway in spermatogonia is not fully clarified. In a murine model, upon deprivation of gonadotropins, the initiation of apoptosis was preceded by p38 MAPK activation and induction of iNOS (66) and this seems to be the case also in normal adult men (51, 52). FSH anti-apoptotic effects seems to occur both in Sertoli cells and in germ cells (64) and, in the latter, both before and after meiosis (49, 50, 64). Interestingly, it has been shown that the mechanisms by which gonadotropins promote the survival of germ cells can be different depending on the cell type (51, 52). In Sertoli cells, FSH promotes anti-apoptotic pathways presumably trough activation of protein kinase B/AKT protein (67). These results suggest that FSH may regulate proliferation and development of male germ cells both indirectly, by acting on Sertoli cells, and directly by up-regulating anti-apoptotic pathways in germ cells. There is also evidence for an effect of FSH on sperm maturation. Baccetti et al. (68) reported an improvement of semen quality and ultrastructural characteristics of spermatozoa in men with high levels of apoptosis and immaturity features in their spermatozoa, supporting the anti-apoptotic and pro-maturation role of FSH in human testis. Recently, a role of FSH favoring sperm maturation has been suggested by the above mentioned study of Casamonti et al. (23), which demonstrated that FSH increases the number of spermatozoa binding to Hyaluronic acid. Although the mechanism(s) through which FSH may promote sperm maturation are mostly unknown, interestingly, a disturbance in the normal replacement of histones by protamines during spermiogenesis, leading to poor condensation of spermatid nuclei, has been demonstrated in FSHR KO mice (69). Sperm maturation is closely linked to DNA integrity. Indeed, it is during spermiogenesis that the replacement of histones with protamines occurs and, as mentioned, a derangement of this process may lead to DNA fragmentation due to lack of re-ligation of the nicks necessary for chromatin compaction (70, 71). In addition, there is evidence that a disturbance of the process of chromatin compaction can represent a trigger for induction of apoptosis in the testis (28). Finally, increased ability of sperm to bind to hyaluronic acid has been associated to higher chromatin compaction and decreased DNA fragmentation (24, 72).

**Figure 1:**
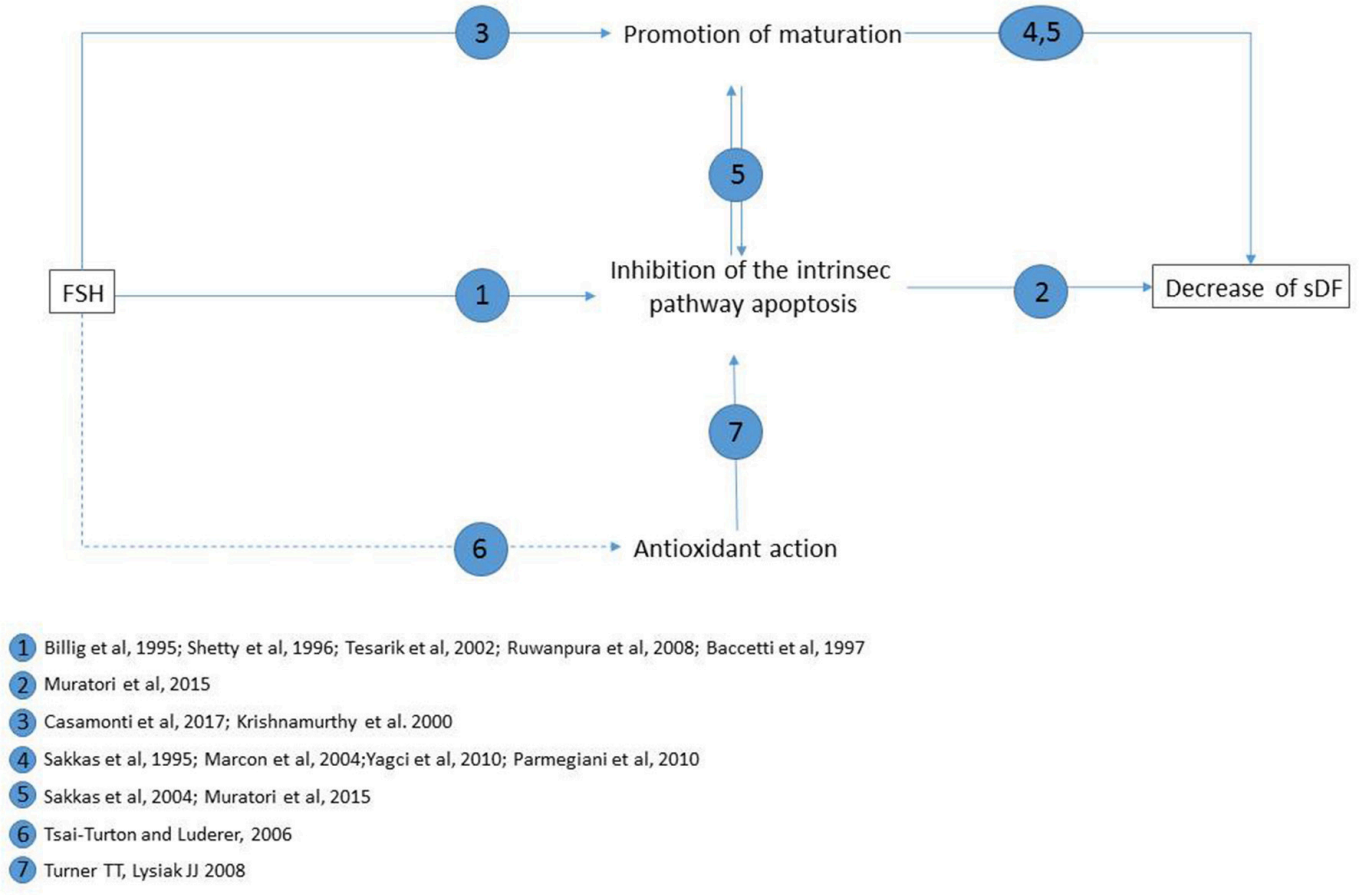
Main possible mechanisms of FSH reducing effect on sperm DNA fragmentation levels, and supporting literature for each pathway. Straight lines: pathways demonstrated in testis or *in vitro* testicular cells; dotted lines: hypothesized pathway (as demonstrated to occur in pre-ovulatory follicules *in vitro*).

As mentioned above, DNA damage can be produced also by a direct attack of ROS. Although, at present, there is no evidence of an anti-oxidant effect of FSH in the testis or in spermatogonial cells *in vitro*, such effect of the hormone cannot be excluded, as it reduces oxidative stress-induced apoptosis in ovarian cells (63). It should be mentioned that oxidative stress may produce the formation of breaks and stable DNA adducts also through a direct attack to DNA (31) and that such damage could persist following FSH treatment. The main possible mechanisms of FSH-related decrease of sDF levels are summarized in Figure 1.

## Conclusion

Although sDF is an important reproductive problem affecting the outcomes of both natural and assisted reproduction, effective treatments to prevent or limit the sperm DNA damage in men are presently scarce. Treatment with FSH appears promising as there is evidence of a beneficial effect of the treatment on sDF (53). However, the lack of clear and univocal patient inclusion criteria contributes to the high heterogeneity of the clinical studies published so far, which does not allow to draw clear-cut conclusions about the effectiveness of the hormone on sperm DNA damage. Future studies should not only include cut-off values of sDF among patient inclusion criteria but also consider the pharmacogenetic evidence of FSH action to identify subjects that may not have beneficial effects from the therapy.

## Author Contributions

All authors listed have made a substantial, direct and intellectual contribution to the work, and approved it for publication.

### Conflict of Interest Statement

The authors declare that the research was conducted in the absence of any commercial or financial relationships that could be construed as a potential conflict of interest.
